# Paracetamol as a Post Prandial Marker for Gastric Emptying, A Food-Drug Interaction on Absorption

**DOI:** 10.1371/journal.pone.0136618

**Published:** 2015-09-09

**Authors:** R. Bartholomé, B. Salden, M. F. Vrolijk, F. J. Troost, A. Masclee, A. Bast, G. R. Haenen

**Affiliations:** 1 Department of Toxicology, Faculty of Health, Medicine and Life Sciences, Maastricht University, Maastricht, The Netherlands; 2 Department of Internal Medicine, Division of Gastroenterology and Hepatology, Maastricht University Medical Center, Maastricht, The Netherlands; University of Pisa, ITALY

## Abstract

The use of paracetamol as tool to determine gastric emptying was evaluated in a cross over study. Twelve healthy volunteers were included and each of them consumed two low and two high caloric meals. Paracetamol was mixed with a liquid meal and administered by a nasogastric feeding tube. The post prandial paracetamol plasma concentration time curve in all participants and the paracetamol concentration in the stomach content in six participants were determined. It was found that after paracetamol has left the stomach, based on analysis of the stomach content, there was still a substantial rise in the plasma paracetamol concentration time curve. Moreover, the difference in gastric emptying between high and low caloric meals was missed using the plasma paracetamol concentration time curve. The latter curves indicate that (i) part of the paracetamol may leave the stomach much quicker than the meal and (ii) part of the paracetamol may be relatively slowly absorbed in the duodenum. This can be explained by the partition of the homogenous paracetamol-meal mixture in the stomach in an aqueous phase and a solid bolus. The aqueous phase leaves the stomach quickly and the paracetamol in this phase is quickly absorbed in the duodenum, giving rise to the relatively steep increase of the paracetamol concentration in the plasma. The bolus leaves the stomach relatively slowly, and encapsulation by the bolus results in relatively slow uptake of paracetamol from the bolus in the duodenum. These findings implicate that paracetamol is not an accurate post prandial marker for gastric emptying. The paracetamol concentration time curve rather illustrates the food-drug interaction on absorption, which is not only governed by gastric emptying.

**Trial Registration:**

ClinicalTrials.gov NCT01335503 Nederlands Trial Register NTR2780

## Introduction

In order to display a systemic biological effect most compounds first have to be absorbed from the gastro-intestinal tract. The onset of the effect depends heavily on the rate of absorption. Absorption mainly takes place once the compound has reached the intestine. Gastric emptying is considered to be the bottleneck in uptake, and numerous studies have been conducted on the effect of nutrition and disease on this process.

Paracetamol is the number one tool for monitoring gastric emptying [1]. To assess gastric emptying, paracetamol is mixed trough a meal. After ingestion, the meal containing paracetamol will enter the stomach, and the meal is digested. Gradually the meal-paracetamol mixture passes through the pyloric sphincter into the duodenum. The large surface area, the peristaltic movement, the relatively thin membrane and the rich blood supply of the intestine enable rapid uptake in the duodenum. It is assumed that paracetamol will be absorbed almost immediately after leaving the stomach. Moreover, it is assumed that the passage time of paracetamol through the stomach is identical to that of the meal. Based on these assumptions, the rate of paracetamol uptake into the plasma would be governed by gastric emptying and consequently gastric emptying might be deduced from the time course of the plasma concentration of paracetamol [2–5].

This approach with paracetamol as marker molecule is regarded as an established and well validated method and is extensively applied. The wide application and acceptance of the procedure, might explain why a critical attitude is lacking. Often, only a small number of samples (sometimes even only one) is taken which will give only a rudimentary kinetic profile. Surprisingly, only a few studies addressed the validity of the method [6, 7]. In the most extensive literature review on the subject is was stated that “it is a simple, noninvasive, and economical method, making it suitable for application on a larger scale but further research should be performed under standardized conditions to allow wide scale clinical use” [6]. Moreover, in a recent study, we obtained data which are in conflict with the widely accepted model which prompted us to carefully evaluate the use of paracetamol as post prandial marker for gastric emptying. This revealed two major pitfalls, i.e. (i) part of the paracetamol may leave the stomach much quicker than the meal and (ii) part of the paracetamol may be relatively slowly absorbed after entering the duodenum.

## Materials and Methods

### Clinical study

In the randomized cross-over study “*Aspergillus niger*-derived enzyme effectively digests gluten in the stomach *of healthy volunteers”* (December 2011 to May 2012) gastric emptying was assessed with the use of paracetamol. The healthy volunteers attended four test days with at least one week washout period between two test days. On two test days the volunteers were given a low caloric meal (143 kcal), administered via a nasogastric feeding tube (Freka Trelumina, Fresenius Kabi Nederland b.v., Zeist, The Netherlands). One of the two low caloric meals given to each of the volunteers was pretreated with ANPEP. On the other two test days a high caloric meal (405 kcal) was administered. Also one of the two high caloric meals given to each of the volunteers was pretreated with ANPEP. The ANPEP treatment had no effect on the paracetamol data and therefor the results of the meal treated with ANPEP were combined with that of the meal that was not treated.

**Fig 1:**
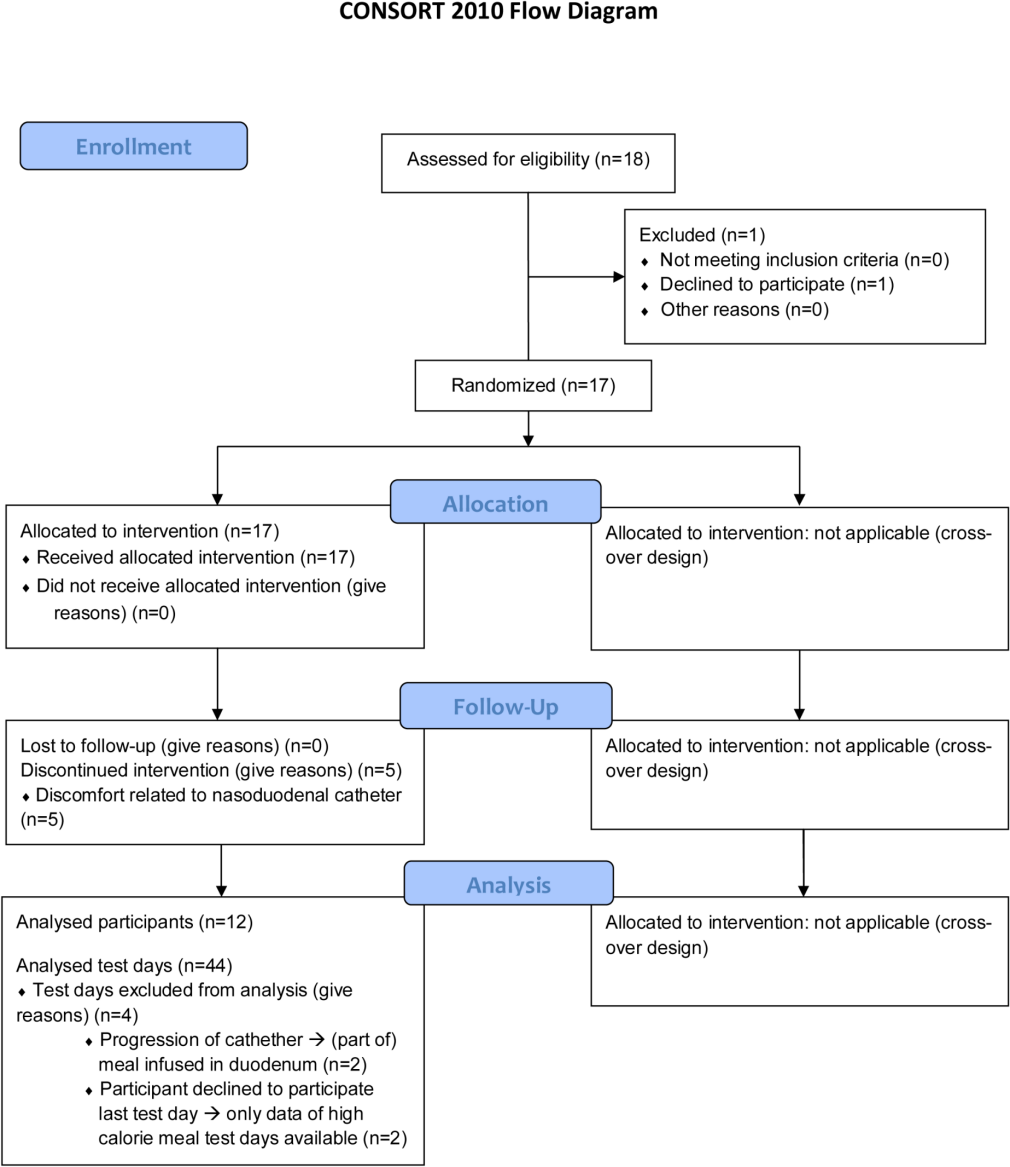
Consort Flow Diagram of the study.

Prior to administration, 1000 mg paracetamol was added to the meals and the meal was thoroughly mixed. The rate of gastric emptying was determined using two different strategies. The common strategy is based on the paracetamol plasma concentration. Blood samples were taken 0, 10, 15, 30, 60, 90, 120, 150, 180, 210 and 240 minutes after administration of the meal. Further details are given in the protocol (S1 Study Protocol ANPEP). The flow diagram of the study is presented in Fig 1.

A total of 12 (8 male and 4 female) healthy volunteers (average age 26 ± 6 years, average BMI 22 ± 3 kg/m^2^) were included in the study. One of these subjects did not complete the fourth test day. In two other subjects the catheter progressed more distally into the small intestine on one occasion (based on the high pH of the sample), indicating that (part of) the meal was directly administered into the duodenum. These experiments were omitted, but the other experiments of these subjects were included in the data analysis. Initially 17 subjects were enrolled in the study, but 5 subjects dropped out due to discomfort related to the nasoduodenal tube. Data of dropouts were omitted from analyses.

The amount of paracetamol taken up on 10 and 240 minutes after intake was derived from the area under concentration-time curve in all subjects. The second strategy to determine gastric emptying was based on the paracetamol concentration in the content of the stomach. Samples were aspirated from the stomach on 15, 30, 45, 60, 75, 90, 120, 150, 180, 210 and 240 minutes after intake. In 6 subjects a complete set of stomach samples could be obtained, and the paracetamol content of the stomach samples of these subjects was determined. Paracetamol metabolites were not determined, because the aim of the study was to evaluate the use of paracetamol as tool for gastric emptying. The paracetamol concentration-time curve derived from the HPLC analysis of the sample was used to determine gastric emptying[8].

The fractionation of paracetamol in the stomach was mimicked by adding 100 μl HCl-solution (0.01 M) to 400 μl of the meal to obtain a pH of 2. After centrifugation the pellet was resuspended in a sodium-phosphate buffer of pH 7.4 and centrifuged again. In both supernatants the paracetamol concentration was determined using HPLC.

The study has been approved by the Institutional Review Board of Maastricht University, and all clinical investigations have been conducted according to the principles expressed in the Declaration of Helsinki. Written informed consent has been obtained from the participants. The study has been registered as NTR2780 in the Dutch trialregister (Nederlands Trialregister).

### Determination of Paracetamol

For the determination of the paracetamol concentration, the samples were deproteinated by adding trichloroacetic acid. After centrifugation (800xg, 5 minutes) the supernatant was analyzed using reversed phase HPLC with UV detection at 250nm. The eluent was a mixture of MilliQ and Acetonitrile (97: 3% v/v). The quality control samples as well as the calibration curves were well within the specifications for pharmacokinetic studies [9]. For each subject and each treatment, an extra control sample taken before the administration of Paracetamol was taken. In none of these control samples interfering peaks were observed. The analyses were performed blinded to the study.

### Statistics

The concentration time curve was statistically evaluated in Prism 6 (GraphPad Software, La Jolla, USA). Differences in pharmacokinetic parameters between groups were analyzed using ANOVA. Post-hoc testing was performed using a paired t-test with Bonferroni correction. A P < 0.05 was considered to be statistically significant.

## Results

A liquid meal was administered as a slurry by a nasogastric feeding tube. Paracetamol was mixed through the meal prior to administration for monitoring gastric emptying. Subsequently, the concentration of paracetamol in plasma was determined post prandial. In six volunteers, also the concentration of paracetamol in the content of the stomach was determined. The results of the analysis are given in the supplementary information.

### Partition of the paracetamol

**Fig 2:**
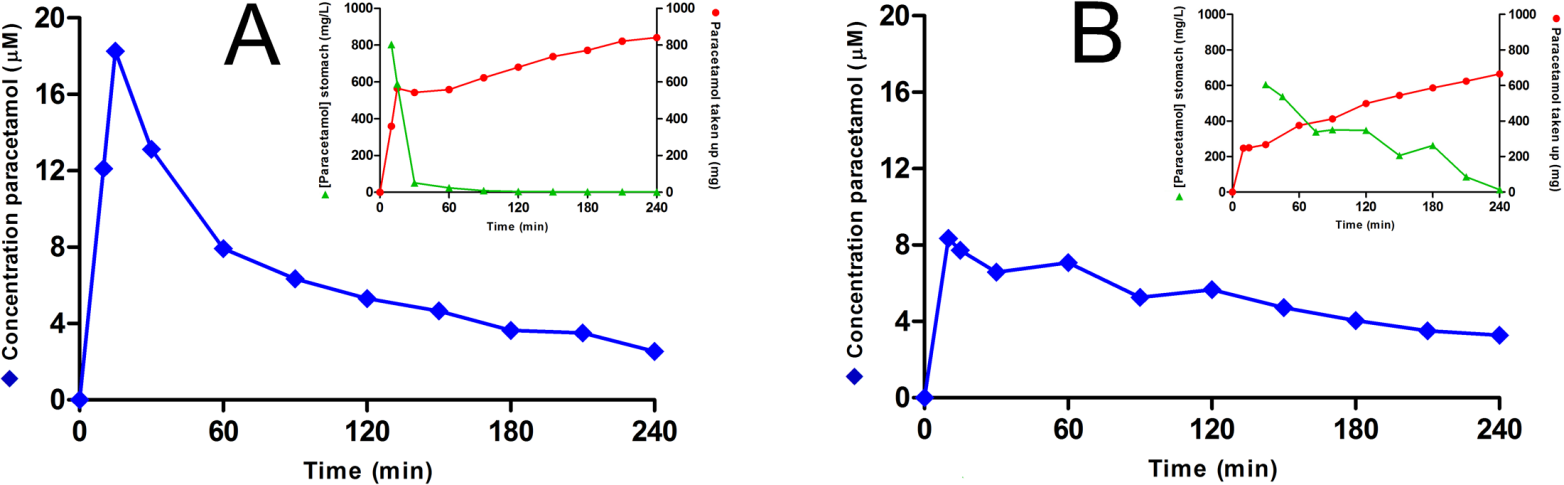
The plasma paracetamol concentration in time after administration of a low or high caloric meal containing 1 g paracetamol via a nasogastric tube. (◆) (Panel A, low caloric meal; panel B, high caloric meal). A typical example is shown. The inserts show the uptake of paracetamol based on the plasma concentration curve (●) and the concentration of paracetamol detected in the stomach content (▲)(panel A, low caloric meal; panel B, high caloric meal).

The paracetamol level in plasma rose after administration of the meal, until it reached a maximum concentration (C_max_) relatively quickly with the low caloric meals. After reaching C_max_, the concentration time curve of paracetamol in plasma showed a biphasic exponential decay. Surprisingly, the exponential decay immediately after reaching C_max_ was higher compared to that at a later stage (p<0,05). An illustration of this kinetic profile is given in Fig 2A.

From the plasma paracetamol data the amount of paracetamol which was taken up was calculated. It appeared that in most subjects, already within 15 minutes, an unexpected high amount of paracetamol was absorbed, even up to 70% of the total dose (Fig 2). The uptake in two phases indicates that paracetamol, administered mixed with the meal, is divided into two fractions. One fraction enters quickly the duodenum and is responsible for the fast uptake. The other fraction apparently stays relatively long in the stomach and is responsible for the relatively slow uptake.

### Encapsulation of part of the paracetamol by the meal.

**Fig 3:**
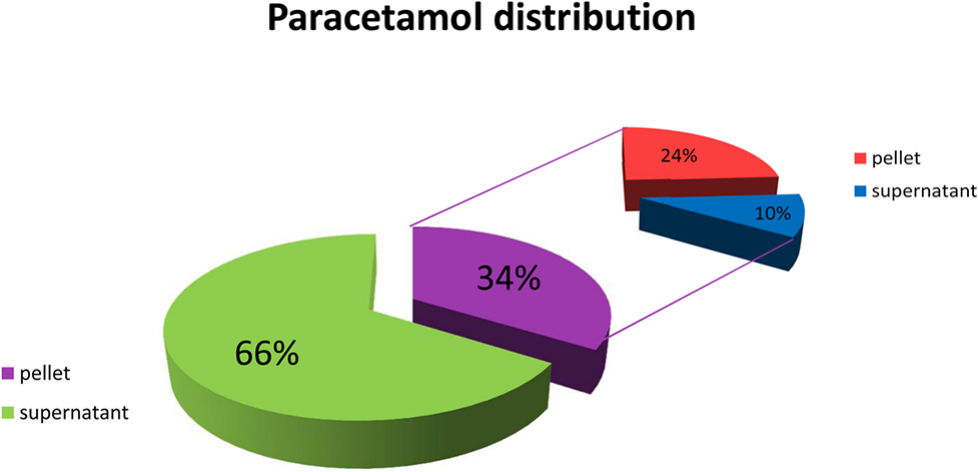
Encapsulation of paracetamol by the meal. The meal containing paracetamol was mixed with a hydrochloric acid solution (pH 2) in a test tube to mimic the conditions in the stomach. After centrifugation two thirds of the paracetamol was retrieved in the supernatant. Extraction of the pellet with buffer (150 mM sodium phosphate buffer, pH 7.4) released one tenth of the paracetamol.

To further investigate the fractionation of paracetamol, a hydrochloric acid solution (to obtain a pH of 2) was added to the low caloric meal containing paracetamol in a test tube to mimic the condition in the stomach. The obtained mixture was centrifuged and two thirds of the paracetamol was retrieved in the supernatant. The pellet was mixed with buffer (150 mM sodium phosphate buffer, pH 7.4) and centrifuged again. This extract of the pellet appeared to comprise approximately one tenth of the original amount of paracetamol (Fig 3). Similar results were obtained with the high caloric meal instead of the low caloric meal (data not shown). These experiment substantiates that in the stomach the paracetamol is divided into two fractions, which are (i) a fraction in which paracetamol is dissolved in the liquid content of the stomach and (ii) a fraction in which paracetamol is encapsulated by the contents of the meal that precipitate in the acid environment of the stomach.

The conclusion is corroborated when the plasma concentration in time is compared to the level of paracetamol detected in the content of the stomach. A slow, but substantial paracetamol uptake in the plasma occurs even when the paracetamol has left the stomach, as shown in Fig 2. This indicates that slow release in the intestine of the paracetamol that is encapsulated by a meal, is a rate-limiting process in the uptake.

### High and low caloric meals

**Fig 4:**
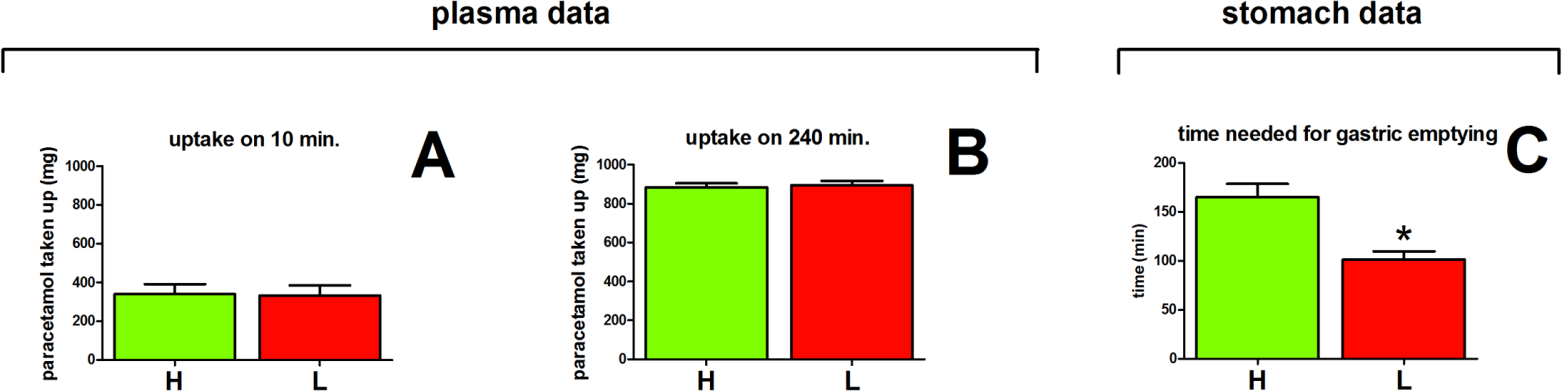
Discrepancy in gastric emptying deduced from the paracetamol plasma data and deduced form content in the stomach, show no difference in gastric emptying between the high and low caloric meals. The amount of paracetamol taken up after 10 min (panel A) and 240 min (panel B) calculated form the concentration time curve of paracetamol in plasma do not show a difference between the high and low caloric meals. However, the time needed for gastric emptying determinded by the paracetamol content of the stomac (panel C), show a clear difference in gastric emptying between the high and low caloric meals. * different form the low caloric value (P < 0.05)

In our study high and low caloric meals were given to the volunteers. The rate of gastric emptying of these meals was estimated from the plasma paracetamol concentration and the concentration of the first time point (10 minutes) was used for this. After 10 minutes a substantial amount of paracetamol, on average 35%, was taken up. No difference was found in the 10 minutes paracetamol concentrations between the low and high caloric meal, suggesting that gastric emptying did not differ between both types of meal (Fig 4A). Also after 240 minutes, the plasma paracetamol data indicate that there is no difference in gastric emptying between the low and high caloric meals (Fig 4B).

The time at which no paracetamol was detected in the stomach content is a direct measure for gastric emptying. These stomach data show that gastric emptying was statistically longer with the high caloric meal compared to the low caloric meal (Fig 4C). The lower gastric emptying of a high caloric meal compared to that of a low caloric meal is well documented, and this difference is confirmed by the longer retention of paracetamol in the stomach samples after the high caloric meal compared to that after the low caloric meal. The difference between the low and high caloric meal is not observed in the gastric emptying determined using the plasma paracetamol concentration (Fig 4).[10]

## Discussion

In the present study the use of paracetamol as tool for gastric emptying is evaluated. The nasogastric feeding tube which was used in our experimental set up, assured that the complete and homogeneous meal/paracetamol mixture was in the stomach at the start of the experiment. After administering the meal, a relatively steep initial increase in the plasma concentration of paracetamol was seen, indicative for a relatively quick uptake. After the quick initial rise, the plasma paracetamol concentration demonstrates a more prolonged, gradual uptake of paracetamol in the blood. Even when all paracetamol has left the stomach, based on analysis of the stomach content, there was still a slow but substantial rise uptake with all meals and in all volunteers. That there still is a substantial uptake of paracetamol when all of the paracetamol has left the stomach already indicates that the plasma paracetamol levels do not only reflect gastric emptying.

The biphasic time course of the paracetamol curve can be explained by the division of paracetamol is divided into two fractions after entering the stomach, a fraction that is encapsulated by the meal and a fraction that is dissolved in the liquid content of the stomach. The aqueous phase is expected to leave the stomach quickly and paracetamol in an aqueous phase is readily absorbed in the duodenum. Deduced from the relatively high and early peak in the plasma paracetamol time course, most paracetamol is in this aqueous phase. This is corroborated by the test tube experiment, whereby acid is added to the meal and only a small part of the paracetamol remains in the solid content of the meal that precipitates. Apparently, the steep initial increase of paracetamol plasma concentration should not be mistaken for a rapid gastric emptying which would give an overestimation of the rate of gastric emptying.

Part of the paracetamol is encapsulated by the meal and this fraction might be used to determine the gastric emptying. However, after all paracetamol has passed the stomach, still a slow uptake of paracetamol in the plasma is detectable. This indicates that the paracetamol is not instantaneously taken up once the meal containing paracetamol reaches the duodenum. Apparently, paracetamol encapsulated by the meal is only slowly released from the bolus and this slow release appears to be the bottleneck in paracetamol uptake in our experiment. This is also corroborated by the test tube experiment, paracetamol encapsulated by the meal after acid precipitation is only partial released by an extraction. The slow release of the encapsulated paracetamol is probably also the result of the relatively high viscosity of the bolus, which reduces the rate of absorption in the intestine [3, 10–12]. In fact, the bioaccessibility, i.e. the release of the compound from the matrix in the intestine, is the rate limiting factor for absorption. This was previously also observed for phenolic compounds in bread [13].

It is well established that the rate of gastric emptying of high caloric meals is slower than that of low caloric meals [14]. This is confirmed by the paracetamol concentration time course in the stomach in our study that does demonstrate a slower rate of gastric emptying for the high caloric meals. However, the plasma paracetamol data fail to reflect this difference in gastric emptying of low and high caloric meals. This confirms that for the meals studied, the plasma paracetamol concentration time curve is not a valid marker for gastric emptying.

Although plasma paracetamol concentrations are an inappropriate measure of the gastric emptying, by definition the paracetamol concentration time curve does reflect the absorption of paracetamol. Actually, our study shows the effect of food on the rate of absorption of paracetamol. The absorption of a drug in the gastrointestinal tract bears major clinical relevance. Often it is advised to take medication in combination with or after a meal to avoid a peak concentration which would prevent side effects and possible toxicity. Our study shows that this is not necessarily the case. In case of enteral feeding still a relatively high peak concentration can arise. Moreover, the duration of the effect might be extended by encapsulation of the drug by the meal and the slow release of the drug from the drug-meal bolus.

Food-drug interactions are not limited to an effect of food on gastric emptying; apparently, it also includes separation of the drug into different fractions, which has an impact on both the onset and duration of the pharmacotherapeutic effect. However, in studying food-drug interactions, it is important to realize that a drug might be separated in two fractions which have a large difference in their rate of absorption. In the use of paracetamol plasma levels as a post prandial marker for gastric emptying, the food-drug interaction on absorption may lead to incorrect conclusions.

## Supporting Information

S1 Composition Meals (XLSX)

S1 Study Protocol ANPEP (DOC)

S1 Study Protocol ANPEP Amendment (DOC)

S1 Trend Statement Checklist (PDF)
