# Supplementary material for: Paracetamol as a Post Prandial Marker for Gastric Emptying, A Food-Drug Interaction on Absorption
Source: PLoS One. 2015 Sep 9;10(9):e0136618. doi: 10.1371/journal.pone.0136618 (PMC4564199; doi:10.1371/journal.pone.0136618)
Supplement: S1 Study Protocol ANPEP — (DOC) [file pone.0136618.s002.doc]

Human Study Protocol

| Title | Effect of AN-PEP enzyme on gastrointestinal breakdown of immunogenic gluten epitopes in healthy subjects |
| --- | --- |

| Study acronym | AN-PEP-02 |
| --- | --- |

| Protocol Number |  |
| --- | --- |

| Version |  |
| --- | --- |

| Date | 03 June 2015 |
| --- | --- |

| Sponsor | DSM Food Specialties B.V.  PO Box 1, 2600 MA Delft  Alexander Fleminglaan 1,  2613 AX Delft  The Netherlands |
| --- | --- |

**Sponsor**

| **Project Manager** | Dr Birgit Schulze |
| --- | --- |
| Company | DSM Food Specialties |
| Postal address | PO Box 1, PP 618-0065, 2600 MA Delft, The Netherlands |
| Visiting address | Alexander Fleminglaan 1, 2613 AX Delft, The Netherlands |
| Phone | +31 15 279 2216 |
| Mobile | +31 6 5156 4745 |
| E-mail | Birgit.Schulze@DSM.com |

| **Responsible Scientist** | Dr Maaike Bruins |
| --- | --- |
| Company | DSM Biotechnology Centre |
| Postal address | PO Box 1, PP 699-0330, 2600 MA Delft, The Netherlands |
| Visiting address | Alexander Fleminglaan 1, 2613 AX Delft, The Netherlands |
| Phone | +31 15 2792056 |
| Mobile | +31 6 13207601 |
| E-mail | Maaike.Bruins@DSM.com |
|  |  |

| **Corporate Scientist** | Prof Dr Wim Saris |
| --- | --- |
| Company | DSM Food Specialties |
| Department | DSM Biotechnology Centre |
| Visiting address | Alexander Fleminglaan 1, 2613 AX Delft, The Netherlands |
| Phone | +31 15 2792056 |
| Mobile | +31 53641475 |
| E-mail | Wim.Saris@DSM.com |

**Principle Investigator**

| **Principle Investigator** | Prof Dr Ad Masclee |
| --- | --- |
| Institution | Maastricht University Medical Center, NUTRIM |
| Department | Department of Internal Medicine, Division of Gastroenterology-Hepatology |
| Postal address | P.O. Box 5800, 6202 AZ Maastricht |
| Visiting address | Debeijeplein 25, 6229 HX Maastricht |
| Phone | +31 43 3875021 |
| E-mail | a.masclee@mumc.nl |

| **Study coordinator** | Dr Freddy Troost |
| --- | --- |
| Institution | Maastricht University, NUTRIM |
| Department | Dept. of Internal Medicine, div. Gastroenterology-Hepatology |
| Postal address | P.O. Box 616, 6200 MD Maastricht |
| Visiting address | Universiteitssingel 50, 6229 ER Maastricht |
| Phone | +31 43-3884296 / 1467 |
| E-mail | f.troost@maastrichtuniversity.nl |

| **Independent physician (onafhankelijk arts)** | Prof Dr H. Schouten |
| --- | --- |
| Institution | Maastricht University Medical Center, NUTRIM |
| Department | Department of Internal Medicine |
| Postal address | P.O. Box 5800, 6202 AZ Maastricht |
| Visiting address | Debeijeplein 25, 6229 HX Maastricht |
|  |  |
|  |  |

**Other Investigators**

| **Investigator** | Prof Dr Frits Koning |
| --- | --- |
| Institution | Leiden University Medical Center |
| Department | Department of Immunohematology & Blood Transfusion |
| Postal address | PO Box 9600, 2300 RC Leiden |
| Visiting address | E3-Q, Gebouw 1, Albinusdreef 2, 2333 ZA Leiden |
| Phone | +31 71 5266673 |
| E-mail | f.koning@lumc.nl |

**Synopsis**

| 1. Title | Effect of AN-PEP enzyme on gastrointestinal breakdown of immunogenic gluten epitopes in healthy subjects |
| --- | --- |
| 1. Location of study site(s) | *Principal investigator: Prof Dr Ad Masclee and Dr Freddy Troost*  Department of Internal Medicine, Division of Gastroenterology-Hepatology, Maastricht University Medical Center, NUTRIM, Maastricht  *Investigator: Prof Dr Frits Koning*  Department of Immunohematology & Blood Transfusion, Leiden University Medical Center, Leiden |
| 1. Background | Coeliac disease is an autoimmune disorder of the small intestine that occurs in genetically predisposed people. Coeliac disease is caused by an immune reaction to gluten protein found in wheat, barley and rye. The immune system cross-reacts with the small-bowel tissue, causing an inflammatory reaction, particularly in the distal duodenum. That leads to a truncating of the villi lining the small intestine and mal-absorption of nutrients. The only available treatment is a lifelong gluten-free diet.  The gluten epitopes that are responsible for the immune reaction, are rich in proline. The DSM enzyme AN-PEP (*Apergillus Niger* Prolyl EndoProtease) specifically cleaves gluten epitopes by cleaving behind these proline-residues. *In vitro,* AN-PEP was shown to effectively degrade gluten epitopes into non-immunogenic fragments under gastrointestinal conditions. It remains to be demonstrated *in vivo* to what extent and how fast AN-PEP can degrade gluten epitopes in the stomach and, how much remaining epitopes enter the small intestine. A second question is whether the caloric density of a meal can influence the efficacy of AN-PEP to degrade gluten by delaying gastric emptying and hence prolonging meal residence time. |
| 1. Rationale for study | Primary:   - Assess effect of AN-PEP on gluten epitope degradation in the duodenum   Secondary:   - Assess effect of AN-PEP on gluten epitope degradation in the stomach - Assess effect of meal caloric content on the efficacy of AN-PEP on gluten epitope degradation in the stomach and duodenum |
| 1. Hypothesis & intended claim | AN-PEP degrades gluten epitopes *in vivo*  AN-PEP helps to degrade gluten epitopes |
| 1. Exclusivity area | In vivo breakdown of gluten |
| 1. Study model | Healthy volunteers. gastroduodenal sampling, stable isotope gastric emptying, dye-dilution technique |
| 1. Study design | A randomized, double-blind, placebo-controlled cross-over study in healthy subjects (n=12 completers). |
| 1. Study intervention | All subjects will come to the study site for 4 test days with one week in between. At test days, a gastro-duodenal catheter will be placed in the stomach and duodenum under x-ray control. Subsequently, subjects randomly receive, in a cross-over fashion, one of the 4 following meals:  1. A low caloric gluten meal together with AN-PEP drink  2. A high caloric gluten meal together with AN-PEP drink  3. A low caloric gluten meal together with placebo drink  4. A high caloric gluten meal together with placebo drink  Meals (300 mL) contain fat, carbohydrate and 5.2 g of gluten powder (4 g gluten protein). Low and high caloric meals differ in fat and carbohydrate and are matched for pH (to avoid difference in AN-PEP activity). The meal is co-infused with 50 mL AN-PEP or placebo into the stomach. Stable ^13^C- labelled octanoic acid is added to the meals to measure gastric emptying rate. |
| 1. Study measurements | Gastric and duodenal fluid is sampled in 15 min aliquots at 3x15 min before (-45 to -30; -30 to -15 ; -15-0 min), and 6x15 min aliquots between 0 to 90 min and 5x30 min aliquots between 90 to 240 min after start of meal intake. During sampling, a dye (PEG 3350) is infused into the duodenum at a constant rate. After reaching steady state the recovery of PEG 3350 is used calculate and quantitate gluten epitope output from the stomach into the duodenum.  Portions of the samples are taken for measurement of gluten epitope concentration (Glia-α9, Glia-α20, LMW glt, HMW glt) by ELISA.  In addition, small portions of these samples are taken for pH and dye concentration measurements.  Before and after meal intake, breath is sampled to determine gastric half emptying time by ^13^CO_2_:^12^CO_2_ in breath every 15-30 min for 240 min |
| 1. primary and secondary endpoints | Intervention study  *Primary endpoint*   1. Effect AN-PEP with a low or high caloric meal on duodenal gluten epitope exposure   *Secondary endpoints*  Effect AN-PEP with a low or high caloric meal on   1. gastric gluten epitope concentrations and AUC over time 2. gastric and duodenal pH 3. gastric half emptying time 4. gastric and duodenal epitope concentrations or absolute amounts over time related to 3 and 4   Difference in means between a low and high caloric meal with AN-PEP in   1. all endpoints mentioned above |
| 1. No of subjects | For an effect-size (difference/SD) of (50%/38%)=1.3, and a power of 90%, n=12 completers are required at α=0.05 (one-sided) for each group |
| 1. Main inclusion- and exclusion criteria | Inclusion  • Male/female  • Age ≥18 but <45 yr  • Hormonal contraceptive treatment  • Subject has read and understood the information provided on the study and given written informed consent  Exclusion  • Any condition that in the opinion of the investigators may interfere with the study and may jeopardise the health status of the participant |
| 1. Investigational product | 6.1 ml AN-PEP (pH 4.2). A 6.1 mL solution consisting of water (79,8 wt%) containing maltodextrin (21,02 wt%), caramel liquid (0,17 wt%), and sodium benzoate (0,36 wt%) at pH 4.2 with a similar appearance as AN-PEP will serve as placebo |
| 1. Safety | Reporting of AE’s and gastrointestinal symptom questionnaire |
| 1. Regulation and guidelines | WMO, declaration of Helsinki, DBC human study SOPs |
| 1. Statistics | Mann-Whitney U and repeated measures procedure |
| 1. Timings | Protocol submission to MEC: April 13, 2011  Last subject out: 31 Oct ‘11  Study report: 31 Dec ‘11 |

**Abbreviations**

AN-PEP Aspergillus Niger Prolyl EndoProtease

AE Adverse events

AUC Area under the curve

CRF Case report form

DFS DSM Food Specialties b.v.

GMP Good Manufacturing Practice

GRAS Generally recognized as safe

GSRS Gastrointestinal symptoms rating scale

MEC Ethical committee (in Dutch: Medische Ethische Toetingscommissie)

NUTRIM School for Nutrition, Toxicology & Metabolism

PEG Polyethylene glycol

SAE Serious adverse events

TIM Gastrointestinal simulation model

**Table of Contents**

[**1** **Introduction 9**](#__RefHeading___Toc286991839)

[1.2 Background information 9](#__RefHeading___Toc286991840)

[1.3 Objective of the study 10](#__RefHeading___Toc286991841)

[2 Sites and Subjects 10](#__RefHeading___Toc286991842)

[2.2 Study site setting 10](#__RefHeading___Toc286991843)

[2.3 Study population 10](#__RefHeading___Toc286991844)

[2.4 Inclusion criteria 10](#__RefHeading___Toc286991845)

[2.5 Exclusion criteria 10](#__RefHeading___Toc286991846)

[3 Study Design and Subject Assignment 10](#__RefHeading___Toc286991847)

[3.2 Design 10](#__RefHeading___Toc286991848)

[3.3 Duration of study 10](#__RefHeading___Toc286991849)

[3.4 Randomization procedure 11](#__RefHeading___Toc286991850)

[3.5 Unblinding procedure 11](#__RefHeading___Toc286991851)

[4 Investigational Product and Dosage Regimen 11](#__RefHeading___Toc286991852)

[4.2 Investigational product 11](#__RefHeading___Toc286991853)

[4.2.1 Product characteristics 11](#__RefHeading___Toc286991854)

[4.2.2 Product safety 11](#__RefHeading___Toc286991855)

[4.2.3 Product formulation 12](#__RefHeading___Toc286991856)

[4.2.4 Packaging and labelling 12](#__RefHeading___Toc286991857)

[4.2.5 Handling and storage conditions 12](#__RefHeading___Toc286991858)

[4.2.6 Handling at the study site 12](#__RefHeading___Toc286991859)

[4.3 Dosage regimen 12](#__RefHeading___Toc286991860)

[5 Study Procedures 13](#__RefHeading___Toc286991861)

[5.2 Recruitment procedure 13](#__RefHeading___Toc286991862)

[5.3 Screening, informed consent, incentive, and withdrawal 13](#__RefHeading___Toc286991863)

[5.3.1 Screening and informed consent 13](#__RefHeading___Toc286991864)

[5.3.2 Incentive 13](#__RefHeading___Toc286991865)

[5.3.3 Withdrawal 13](#__RefHeading___Toc286991866)

[5.4 Intervention 13](#__RefHeading___Toc286991867)

[5.4.1 Gastroduodenal catheter 13](#__RefHeading___Toc286991868)

[5.4.2 Intervention 14](#__RefHeading___Toc286991869)

[5.4.3 Test meals 14](#__RefHeading___Toc286991870)

[5.5 Sample taking 15](#__RefHeading___Toc286991871)

[5.5.1 Gastroduodenal sampling 15](#__RefHeading___Toc286991872)

[5.5.2 Breath sampling 15](#__RefHeading___Toc286991873)

[5.6 Schedule of assessments 15](#__RefHeading___Toc286991874)

[6 Sample analyses 16](#__RefHeading___Toc286991875)

[6.2 Analyses and calculations 16](#__RefHeading___Toc286991876)

[6.2.1 Epitopes 16](#__RefHeading___Toc286991877)

[6.2.2 Volume marker 16](#__RefHeading___Toc286991878)

[6.2.3 Breath samples 16](#__RefHeading___Toc286991879)

[7 Statistical Plan 16](#__RefHeading___Toc286991880)

[7.2 Endpoints 16](#__RefHeading___Toc286991881)

[7.2.1 Primary endpoint 16](#__RefHeading___Toc286991882)

[7.2.2 Secondary endpoints 16](#__RefHeading___Toc286991883)

[7.3 Calculations 17](#__RefHeading___Toc286991884)

[7.3.1 Area under the curve 17](#__RefHeading___Toc286991885)

[7.3.2 Absolute amounts 17](#__RefHeading___Toc286991886)

[7.4 Statistical methods 17](#__RefHeading___Toc286991887)

[7.5 Sample size calculation 17](#__RefHeading___Toc286991888)

[7.6 Subject population(s) for analysis 17](#__RefHeading___Toc286991889)

[7.7 Termination Criteria 17](#__RefHeading___Toc286991890)

[8 Safety and reporting 18](#__RefHeading___Toc286991891)

[8.1 Health condition and adverse event reporting 18](#__RefHeading___Toc286991892)

[8.2 Serious adverse event reporting 18](#__RefHeading___Toc286991893)

[9 Ethical Responsibilities 18](#__RefHeading___Toc286991894)

[9.2 Study conduct 18](#__RefHeading___Toc286991895)

[9.3 Declaration of Helsinki 18](#__RefHeading___Toc286991896)

[9.4 Ethics committee approval 18](#__RefHeading___Toc286991897)

[9.5 Insurance of participants 19](#__RefHeading___Toc286991898)

[9.6 Subject information and obtaining consent 19](#__RefHeading___Toc286991899)

[9.7 Withdrawals of subjects 19](#__RefHeading___Toc286991900)

[9.8 Subject confidentiality 20](#__RefHeading___Toc286991901)

[9.9 Benefit and risk assessment 20](#__RefHeading___Toc286991902)

[10 Data Handling and Record Keeping 21](#__RefHeading___Toc286991903)

[10.2 Direct Access to Source Data/Documentation 21](#__RefHeading___Toc286991904)

[10.3 Storage of data and documents 21](#__RefHeading___Toc286991905)

[11 Administrative Aspects and Reporting 21](#__RefHeading___Toc286991906)

[11.2 Amendments 21](#__RefHeading___Toc286991907)

[11.3 Annual progress report 21](#__RefHeading___Toc286991908)

[11.4 End of study report 21](#__RefHeading___Toc286991909)

[12 References 22](#__RefHeading___Toc286991910)

# Introduction

## Background information

Coeliac disease is an autoimmune disorder of the small intestine that occurs in genetically predisposed people. Coeliac disease is caused by an immune reaction to gluten protein found in wheat, barley and rye. The immune system cross-reacts with the small-bowel tissue, causing an inflammatory reaction, particularly in the distal duodenum. That leads to a truncating of the villi lining the small intestine and mal-absorption of nutrients. The only known effective treatment is a lifelong gluten-free diet. The gluten epitopes that are responsible for the immune reaction are rich in proline residues.

The DSM enzyme AN-PEP (*Aspergillus Niger* Prolyl EndoProtease, also known as *Tolerase*) specifically cleaves gluten epitopes by cleaving behind these proline-residues. Proteases are widely present in plants and mushrooms. Prolyl endoproteases are naturally present in food such as edible mushrooms (Sattar, J Biochem, 1990) and spinach (Kuwabara, FEBS Lett, 1992). Proteases have also been used in food processing for centuries. The major protease employed in the food industry includes chymosin for cheese production.

Protein digesting enzymes are secreted by the pancreas and are present in the small intestine. Various digestive proteolytic enzymes are being sold as dietary supplements to support digestion for example for people with pancreatic insufficiency. These supplements typically contain lipases and proteases. These supplements have been used for many years efficaciously and with no side effects (Cerf-Bensussan, Gut, 2007). Also, a number of protease-containing dietary supplements for gluten digestion are on the market. Examples are *Glutenzyme Plus* from Biocare, containing, amongst others, cellulase, protease and amylase. *Glutenzyme* from Pharmax contains a mixture of proteases. The supplements *Glutenase Plus* (Immunecare) and *Gluten Digest* contain Biocore® dipeptidyl peptidase. Dipeptidyl peptidase, like AP-PEP, cleaves behind protein proline residues but has a neutral pH optimum being less effective in degrading gluten in the stomach.

*In vitro* it was shown that AN-PEP effectively degrades gluten epitopes into small non-immunogenic fragments under gastrointestinal conditions. It remains to be demonstrated *in vivo* to what extent and how fast AN-PEP can degrade gluten epitopes in the stomach and, how much remaining epitopes enter the small intestine. A second question is whether the caloric density of a meal can influence the efficacy of AN-PEP to degrade gluten by delaying gastric emptying and hence prolonging meal residence time.

DFS started to investigate this enzyme, as a digestive aid to help degrading gluten. AN-PEP has been investigated in a number of *in vitro* studies and one human pilot study with coeliac patients. AN-PEP was shown to effectively degrade gluten epitopes *in vitro* (Stepniak Am J Physiol Gastrointest Liver Physiol. 2006). In the TNO gastrointestinal simulation model (TIM), AN-PEP degraded immunogenic gluten epitopes of a hamburger roll when given as Big Mac menu (Mitea Gut 2008). This indicates that AN-PEP digests gluten epitopes even in the presence of a complex food matrix. Between May 2008 and April 2009, DFS conducted a randomised, double-blind, placebo-controlled parallel study with coeliac disease patients. The aim of this proof-of-concept study was to assess the safety and efficacy of AN-PEP compared to control in patients with celiac disease consuming gluten. In this study, AN-PEP appeared to be safe; no serious adverse events or any adverse reactions were reported. However, unexpectedly, only few patients responded clinically to the 2-wk gluten intake and, therefore, a reducing effect of AN-PEP on the clinical response to gluten was not assessable. This study has now been submitted for publication (submission enclosed as appendix K4 to this study protocol, under confidentiality). Before the potential efficacy of AN-PEP to reduce clinical symptoms will be evaluated again in a follow-up study, the feeding regime will have to be optimized. In the current study, the efficacy of AN-PEP administered during consumption of different meals will be investigated in healthy volunteers.

## Objective of the study

- Primary: demonstrate the in vivo efficacy of AN-PEP on gluten epitope degradation
- Secondary: demonstrate whether caloric content of a meal affects AN-PEP efficacy

# Sites and Subjects

## Study site setting

Monocenter study in hospital setting: all subjects will be recruited at the Maastricht University and Maastricht University Medical Centre

## Study population

Seventeen healthy male or female subjects

## Inclusion criteria

- Male/female
- Age ≥18 but <45 yr
- Subject is willing to undergo all protocol related assessments
- Subject has read the information provided on the study and given written consent
- Women who are treated with hormonal contraceptives

## Exclusion criteria

- Any medical condition that in the opinion of the investigators may interfere with the study and may jeopardise the health status of the participant.
- Pregnancy or wish to become pregnant during the course of this experiment.
- Women who are not treated with hormonal contraceptives

# Study Design and Subject Assignment

## Design

Double-blind, placebo-controlled, randomized, crossover design

Subjects attend to four test days during the study period with one-week washout period in between. Each test day they receive one of four interventions: a low caloric gluten meal with AN-PEP or with placebo or a high caloric meal with AN-PEP or with placebo.

## Duration of study

Subject recruitment will start after MEC approval of the study.

Intervention study: after obtaining signed informed consent and inclusion, each subject will have four test days at the study site with one-week washout period in between. Hence, each subject will participate during 3 weeks in the study. In total, 12 subjects will complete the study. To anticipate on possible drop-out during the study due to unforeseen reasons, such as illness, a total of 17 subjects will be recruited. Assuming that each week four volunteers are willing to participate and can be tested, the total study test duration is 12 weeks. Assuming some subject withdrawals, the total study duration will be 4.5 months.

## Randomization procedure

At test days, subjects will be randomised in a double-blind fashion in blocks of four, to one of the following meals:

1. A low caloric gluten meal together with AN-PEP drink
2. A high caloric gluten meal together with AN-PEP drink
3. A low caloric gluten meal together with placebo drink
4. A high caloric gluten meal together with placebo drink

DFS will label the AN-PEP or placebo tubes by A and B according to the randomisation schedule. The randomisation list will be generated by the statistician of DFS using a computerised procedure. The investigator has no access to the randomization list that conceals the treatment code. The investigator receives sealed code envelopes, which reveal the treatment in case of emergency. The *product numbers* as allocated by DFS are registered by the investigator in the screening and enrolment log.

High and low caloric meals are not blinded.

## Unblinding procedure

DFS will prepare for the investigator sealed code envelopes for each subject enrolled into the study identifying which product the subject receives. In general, there should be no need to unblind the allocated product. Unblinding should only be done in those rare cases when a subject suffers from a SAE and the investigator believes that clinical management depends importantly on whether the subject received the product or placebo. If the investigator needs to break the code, DFS (Maaike Bruins or Wim Saris) should, if possible, be contacted prior to breaking the code. The investigator, time, date and reason for breaking the code must be recorded in the SAE form. In all cases, DFS must be notified within 24 hours after the code has been broken.

# Investigational Product and Dosage Regimen

## Investigational product

### Product characteristics

AN-PEP actively cleaves gluten under gastric conditions. The pH optimum of AN-PEP is at 4.5 (between pH 3-6) and AN-PEP is resistant to low pH and to degradation by gastric enzymes. Having an enzyme active under gastric conditions is of foremost importance, as the gluten should already be degraded in the stomach and proximal duodenum since they exert their immune response in the distal duodenum.

### Product safety

Details on the safety of AN-PEP are provided in the attached documents D4 ‘Memo safety statement AN-PEP’ and ‘D4 Safety Tolerase REG statement’. Enzymes in general are naturally present in many food products of the human diet. Many enzyme preparations are also added to food products as processing aids. AN-PEP has a GRAS status. The safety of the AN-PEP enzyme preparation has been confirmed with several toxicity studies (dossier at DFS). AN-PEP consumption also appeared safe in a recent trial in which celiac patients consumed gluten with AN-PEP or placebo for two periods of two weeks; no serious adverse events or any adverse reactions were reported This study has now been submitted for publication (Tack et al, submission enclosed as appendix K4 to this study protocol, under confidentiality). Therefore, it can be concluded that consumption of AN-PEP is not likely to cause adverse effects under the conditions of intended use as proposed in this human trial with healthy subjects. See also the attached document ‘D4 Memo safety statement AN-PEP’.

### Product formulation

The AN-PEP enzyme is produced at an enzyme manufactory in France under food grade conditions (see appendices ‘D4 Statement enzyme food grade compliance’ and ‘D4 Food Grade Material preparation’). The AN-PEP and placebo will be prepared according to Hygienic House Rules with food grade ingredients at the food grade pilot plant at DFS in Delft (see also appendix ‘D4 Food grade facilities statement’.

Powder meals contain food grade ingredients, which are weighted for single portion sachets at the food grade pilot plant at DFS.

### Packaging and labelling

Labels of the AN-PEP and placebo-containing tubes contain information on the storage conditions, study reference, expiry date, product code (A or B), and the respective code number (to trace the batch number).

Dry meals are packaged in sachets of air-tight aluminium foil and labels contain information on the storage conditions, study reference, expiry date, and are labelled “high or low caloric meal” (see appendix D3 ‘Labels’).

The AN-PEP and placebo as well as the dry meals are packaged and labelled at the DFS food grade pilot plant.

### Handling and storage conditions

The AN-PEP, placebo, and dry meals are released by DSM after a quality and safety assessment, which includes microbial analysis and AN-PEP protease activity analysis (which will also be measured bi-monthly throughout the trial).

The AN-PEP and placebo-containing tubes will be stored and shipped at 4°C. Dry meals sachets are stored and shipped at room temperature.

### Handling at the study site

At test days, AN-PEP and placebo drinks and meals are further prepared at the food-grade kitchen facility of NUTRIM (room 3247, Universiteitssingel 50, Maastricht). AN-PEP and placebo are dissolved in a total volume of 100 mL tap water of 40°C by stirring (final temperature 37°C). The dry meal powders are dissolved in a total volume of 300 mL tap water of 40°C by stirring with a spoon and by subsequently mixing with a mixer for less than 2 seconds (final temperature 37°C). For the measurement of gastric emptying rate, 100mg of octanoic acid-1-13C (Campro Scientific, Veenendaal, The Netherlands) is added to the test meal.

## Dosage regimen

It is assumed that an average gluten snack contains approximately 4 g of gluten protein. Based on previous studies, it is assumed that 20 PPU AN-PEP is needed to degrade 1 g of gluten. To degrade 4.8 g of protein (4 g of gluten protein + 0.8 g of caseinate), 6,1 mL of AN-PEP (96 PPU) in a total of 100 mL water will be given with 300 mL of the gluten meal.

A 6.1 mL solution consisting of water (79,8 wt%) containing maltodextrin (21,02 wt%)(FrieslandCampina Kievit B.v., Meppel, The Netherlands), caramel liquid (0,17 wt%) (Syral, Wondelgem, Belgium), and sodium benzoate (0,36 wt%) (Brenntag, Deerlijk, Belgium) at pH 4.2 with a similar appearance as AN-PEP will serve as placebo.

# Study Procedures

## Recruitment procedure

Subjects will be recruited by university advertisements (Appendix E3. *Advertentie*). Participants can contact the investigator for information. Participants expressing their interest will receive an information letter. If the participants are interested in participating, an appointment will be made for the screening day.

## Screening, informed consent, incentive, and withdrawal

### Screening and informed consent

If the subject fulfils the in and exclusion criteria and the participant agrees to participate, he/she will sign an informed consent form. Before giving written informed consent and after providing detailed information on this study orally as well as in writing, subjects will have at least one week to consider participation. At the subsequent screening day, a brief health history and demographic information will be collected in the subject’s case report file (CRF). The CRF does not contain any reference to data that can relate to the individual; only the subject code is indicated on the CRF. The code is kept by the investigator, and is only accessible to the investigator and the principle investigator. For women, a pregnancy test is an obligated part of the screening procedure.

### Incentive

For each test day that volunteers complete, they receive reimbursement of €100. If there is a medical reason to withdraw the subject from the study (as judged by the investigator), subjects receive reimbursement for the test day of withdrawal. Travel costs will ~~not~~ be reimbursed.

### Withdrawal

Any of the earlier-mentioned conditions may lead to premature withdrawal of subjects from the study. All subjects that withdraw and the reason for withdrawal will be documented in the screening and enrolment log. Subjects have the right to withdraw from the study without explaining the reason for this decision.

## Intervention

### Gastroduodenal catheter

Subjects are not allowed to consume any alcohol containing beverages, and abstain from physical exercise on the day prior to testing. The subjects will arrive at the laboratory at 8.00h after an overnight fast (no food or drinks allowed since 10.00 PM, except water *ad libitum*.

At the start of the experiment, a commercially available nasogastric intraduodenal feeding catheter (Freka Trelumina, Fresenius Kabi Nederland b.v., Zeist, The Netherlands) will be inserted by a gastroenterologist. Under intermittent fluoroscopic control, the catheter will be placed with the tube tip located in the duodenum, according to the manufacturers instructions (see manual feeding tube, attached to this protocol). This catheter will be used to administer the test meal into the stomach, and to continuously inject the inert dilution marker polyethylene glycol 3350 (PEG3350) into the proximal duodenum. The catheter will also be used to collect stomach- and intraduodenal fluid by aspiration using a standard 20-ml syringe.

### Intervention

The 300 mL meal (37°C) will be mixed with the 100 mL AN-PEP solution, as was described in section 4.1.4 ‘Packaging and labelling’, stirred and directly infused into the stomach via the intragastric catheter port over a 5-min period, at a rate of 80 mL/min.

Polyethylene glycol 3350 (PEG-3350; Movicolon, Norgine b.v., Amsterdam, The Netherlands; see the attached document D4 SPC Movicolon [Norgine]) is continuously infused at 3 ml/min, in a concentration of 15 mg/mL,via the catheter into the duodenum. PEG-3350 starts exactly 60 min prior to meal infusion, to achieve steady state conditions in fluid secretion and absorption at the moment of meal infusion. PEG-3350 is used as a dilution marker, to calculate fluid dilution by secretions in the bowel (Vu et al, 1999, Lam et al, 1999, Beglinger et al, 1985).

The order at which subjects will receive a low- or a high caloric meal with AN-PEP or placebo, respectively, will be determined during the randomisation procedure (see section 3.4).

Prior to and after meal/AN-PEP administration, gastric and duodenal aspirations, and breath samples will be collected according to the sampling scheme (see section 5.5.1 below).

After finishing the test day, subjects are requested to complete a gastrointestinal symptoms questionnaire, which contains the gastrointestinal symptoms rating scale (GSRS) and an additional symptoms questionnaire according to the *Schedule of Assessments* (section 5.6). The questionnaires are provided in appendix F1 *vragenlijsten*.

### Test meals

5.2 g of gluten powder (77% protein; 4 g of gluten protein) is premixed in all test meals. The low and high caloric meals differ in fat and carbohydrate content (in a constant ratio of 0.7 fat : 1 carbohydrate) but are matched for (low) protein content (to avoid unequal competition with gluten for enzyme). Stable octanoic acid-1-13C (Campro Scientific, Veenendaal, The Netherlands) is added to test meals to measure gastric emptying rate, identical to a recently finished human intervention study that was previously approved by the METC AzM/UM (MEC 09-2-042/ NL27585.068.09). Meals are matched at pH 6 (to avoid difference in AN-PEP activity) by citric acid.

## Sample taking

### Gastroduodenal sampling

Gastric and duodenal content is sampled through a the multilumen nasoduodenal catheter starting before (-45 to -30; -30 to -15 ; -15-0 min), and 6x15 min aliquots between 0 to 90 min after start of meal infusion. Gastric content was also aspirated at t=5 and t=10 minutes. On each sampling occasion, approximately 3 mL was aspirated from the gastric port for pH and gluten epitopes 2 mL from the duodenum for gluten epitopes and pH measurements. After the last meal infusion and after each sample, the catheter will be flushed with 5 mL saline.

| min | -45 | ‑30 | ‑15 | 0 | 5 | 10 | 15 | 30 | 45 | 60 | 75 | 90 | 120 | 150 | 180 | 210 | 240 |
| --- | --- | --- | --- | --- | --- | --- | --- | --- | --- | --- | --- | --- | --- | --- | --- | --- | --- |
| M |  |  |  | X |  |  |  |  |  |  |  |  |  |  |  |  |  |
| A |  |  |  | X |  |  |  |  |  |  |  |  |  |  |  |  |  |
| G | X | X | X |  | X | X | X | X | X | X | X | X | X | X | X | X | X |
| D | X | X | X |  |  |  | X | X | X | X | X | X | X | X | X | X | X |
| F | X | X | X |  |  |  | X | X | X | X | X | X | X | X | X | X |  |
| B |  |  | X |  |  |  | X | X | X | X | X | X | X | X | X | X | X |

M=Meal infusion

A=AN-PEP infusion

G=Gastric samples (pH, gluten)

D=Duodenal samples (pH, PEG, gluten)

F=Flushing catheter

B=Breath samples

Gastric and duodenal samples for gluten epitope measurements are immediately stored liquid nitrogen to stop enzymatic activity and subsequently stored at ‑80°C until analysis. Samples will be shipped on dry ice to the LUMC for gluten epitope measurements.

### Breath sampling

Before and after meal infusion, breath samples (^13^C octanoic acid) are taken to determine gastric half-emptying time by ^13^CO_2_:^12^CO_2_ in breath, every 15-30 min for 240 min (see sampling scheme).

## Schedule of assessments

| Day | 0 | 1 | 2 | 3 | 4 |
| --- | --- | --- | --- | --- | --- |
| Study Information | X |  |  |  |  |
| Eligibility screening | X |  |  |  |  |
| Informed consent | X |  |  |  |  |
| Demographics, medical history | X |  |  |  |  |
| Randomization |  | X |  |  |  |
| Catheter placement |  | X |  |  |  |
| High / low caloric diet |  | X | X | X | X |
| AN-PEP / placebo |  | X | X | X | X |
| Gastrointestinal symptom questionnaire |  | X | X | X | X |
| Gastroduodenal sampling |  | X | X | X | X |
| Breath test |  | X | X | X | X |

The catheter will be removed after completion of this sampling scheme. A new catheter will be placed prior to each experiment.

# Sample analyses

## Analyses and calculations

### Epitopes

Gluten epitope concentrations (Glia-α9, Glia-α20, LMW glt, HMW glt) are measured by ELISA (soluble gluten) and Western blotting (insoluble gluten) (Mitea et al. Gut, 2008).

### Volume marker

PEG-3500 is infused into the duodenum. PEG concentrations are determined by HPLC analysis. It will be used to calculate the dilution of duodenal samples by endogenous secretions, using the formulas described by Beglinger et al; V=(Fx[PEG]perfx10/(PEGmeas), where V is calculated duodenal volume (ml/15 min); F is the flow rate of PEG solution perfused; PEG perf is the concentration of PEG in the perfusate, and PEG meas is the concentration of PEG in the duodenal juice collected for 15 min.

### Breath samples

Gastric emptying will be determined using the C13 stable isotope breath test. The octanoic acid-1-13C breath test is a reliable and non-invasive tool for the analysis of gastric emptying rates of liquid phases without radiation exposure. For this purpose, 100mg of octanoic acid-1-13C (Campro Scientific, Veenendaal, The Netherlands) is added to the test meal ingested at t=0 min. One basal breath sample will be taken 10 min (t=-10 min) before consuming the meal containing the 13C-octanoic acid, subsequent samples are taken at 15-min interval from t=15min the first two hours and at 30-min intervals thereafter until t=240 min. Breath samples for 13CO2 enrichment will be collected using re-usable plastified aluminium bags. Samples will be collected, stored and afterwards analysed using Isotope Ratio Mass Spectometry (Finnigan MAT 252). Half time of gastric emptying will be calculated after curve fitting using methods described by Braden et al.

# Statistical Plan

## Endpoints

### Primary endpoint

Difference in means between AN-PEP and placebo in the low and high caloric meals in

1. duodenal gluten epitope exposure (AUC)

### Secondary endpoints

Difference in means between AN-PEP and placebo with a low or high caloric meal in

1. gastric gluten epitope exposure (AUC)
2. gastric and duodenal epitope concentrations or absolute amounts over time
3. gastric and duodenal pH
4. gastric emptying time
5. gastric and duodenal epitope concentrations or absolute amounts over time related to 4 and 5

Difference in means between the low and high caloric meal with or without AN-PEP in

1. all endpoints mentioned above

## Calculations

### Area under the curve

The 240-min Area Under the Curve of epitope concentration above cut-off levels over time is calculated in SAP using curve fitting.

### Absolute amounts

The dilution of duodenal fluid samples by secretions in the bowel will be determined using the PEG method as described in section 6.2.2 ‘volume marker’. From this dilution, the absolute amounts of gluten epitopes appearing in the duodenum are calculated.

## Statistical methods

The statistical significance of the differences observed between the AUC scores will be assessed by using the non-parametric Mann-Whitney U test at α=0.05. Gastric and duodenal epitope concentrations over time between groups are assessed using one-sided repeated measurements ANOVA (SAS PROC MIXED) using baseline epitope concentration as covariates and time (day) as categorical variable.

Any deviation(s) from the original statistical plan are described and justified in the final report.

## Sample size calculation

We calculated that a sample size of 12 subjects would be required based on a standardized effect-size of 1.3, a power of 90%, and α=0.05 (one-sided). Seventeen subjects were recruited taking into account a drop-out of 5 subjects.

## Subject population(s) for analysis

All analyses will be done on the per-protocol population, i.e. only those subjects who complete the entire trial are counted towards the final results.

## Termination Criteria

The study is closed when 12 subjects have completed the study

# Safety and reporting

## Health condition and adverse event reporting

At the eligibility screening visit, the investigator will report information on the subject’s general health history in the subject’s CRF. Subjects are supervised during the study days and have access to the independent physician during the entire study period.

Adverse events (AEs) are established by the investigator on basis of:

1. Answer to the open question: “How are you feeling?” (AE form)

2. Spontaneous reporting (AE form)

3. Gastrointestinal symptom questionnaire

Subjects will be advised that they should report at their study visits all AEs occurring after informed consent up to two days after study cessation to the investigator who will document them in the CRF and in the AE form. On test days, subjects will also be asked to fill in a gastrointestinal symptom questionnaire to ensure that all gastrointestinal complaints are reported to the investigator.

All adverse events will be followed until they have abated, or until a stable situation has been reached. Depending on the event, follow up may require additional tests or medical procedures as indicated, and/or referral to the general physician or a medical specialist.

## Serious adverse event reporting

All serious adverse events (SAEs) will be reported within 24 hrs to the sponsor, and through the web portal ToetsingOnline to the accredited METC that approved the protocol, within 15 days after the sponsor has first knowledge of the serious adverse reactions, according to the requirements of the METC.

If a SAE occurs, the subject will be followed until the SAE outcome has been established or the condition is stabilised.

# Ethical Responsibilities

## Study conduct

It is the responsibility of the investigator(s) to conduct the study according to the protocol and to ensure that they have the subjects available to conduct the study within the period defined in the study protocol.

## Declaration of Helsinki

The study will be conducted according to the principles of the Declaration of Helsinki (as amended in Tokio, Venice, Hong Kong, Somerset West, Edinburgh, Seoul. Note of clarification added in Washington and Tokio) and in accordance with the Medical Research Involving Human Subjects Act (WMO)

## Ethics committee approval

The principal investigator will submit the study protocol on behalf of DFS. The MEC approval is obtained before the study is started. The approval of the MEC will be sent in writing, to the principal investigator who in turn provides DFS with a copy.

## Insurance of participants

The sponsor has a liability insurance which is in accordance with article 7, subsection 6 of the WMO.

The sponsor (also) has an insurance which is in accordance with the legal requirements in the Netherlands (Article 7 WMO and the Measure regarding Compulsory Insurance for Clinical Research in Humans of 23th June 2003). This insurance provides cover for damage to research subjects through injury or death caused by the study.

1. € 450.000,-- (i.e. four hundred and fifty thousand Euro) for death or injury for each subject who participates in the Research;
2. € 3.500.000,-- (i.e. three million five hundred thousand Euro) for death or injury for all subjects who participate in the Research;
3. € 5.000.000,-- (i.e. five million Euro) for the total damage incurred by the organisation for all damage disclosed by scientific research for the Sponsor as ‘verrichter’ in the meaning of said Act in each year of insurance coverage.

The insurance applies to the damage that becomes apparent during the study or within 4 years after the end of the study.

## Subject information and obtaining consent

The investigator will obtain informed consent from each subject participating in the study, after explanation of the aims, methods, benefits and potential hazards of the study and the insurance coverage. The consent must be obtained before any study-specific procedures are performed. Before giving written informed consent and after providing detailed information on this study orally as well as in writing, subjects will have at least one week to consider participation. Only subjects who are able to give legal consent will be entered into the study. Subject will be explained that they are free to refuse to participate in the study, or that they can withdraw their consent at any time and for any reason, without incurring any penalty or withholding of treatment on the part of the investigator. Subjects have the right to withdraw from the study without explaining the reason for this decision.

Subjects can contact an independent medical doctor to ask questions related to this study. the medical doctor, prof.dr. Schouten, is not associated with this investigaon by any means.

## Withdrawals of subjects

Subjects can leave the study at any time for any reason if they wish to do so without any consequences. The responsible investigator can also withdraw a subject if continuing participation is in his/her opinion deleterious for the subject’s well being. Subjects can also be withdrawn in case of protocol violations and non-compliance. Withdrawals due to non-attendance must be followed up to obtain the reason for non-attendance. When a subject withdraws from the study for a medical reason, volunteers will be approached if they wish to participate in a medical examination. Volunteers retain the right to decline to participate in this examination. Subjects also retain the right to obtain another medical opinion or change physician. In case of withdrawal because of a severe or serious adverse event (SAE), haematological, blood chemistry and urine laboratory tests or other special examinations will be performed. Withdrawals due to intercurrent illness or adverse events must be fully documented in the CRF, with supplementary information where available and/or appropriate. Subjects have the right to withdraw from the study without explaining the reason for this decision.

## Subject confidentiality

The investigator(s) will ensure that the subject's anonymity towards DFS and other third parties will be maintained. On all documents that are to be submitted to DFS, subjects must be identified only by a study number – not by their names or hospital/clinical number. The investigator(s) should keep a separate list which matches the study numbers with the subject's names.

## Benefit and risk assessment

During the test days visits, a catheter will be placed into the stomach and duodenum. There is a very small risk of bowel perforation, but no literature is available on the actual risk. This risk is generally considered as nil. The subjects will perceive mild discomfort during the placement of the catheter. The radiation exposure during the positioning of the feeding tube is minimal. The total exposure to radiation (during all test days) will be approximately 0.20 mSv, which equals the radiation, which is received during a three-hr flight in an aeroplane at a 4-km altitude (www.nrg-nl.com).

AN-PEP can be consumed safely by humans (see document D4 ‘Memo safety statement AN-PEP’).

The high and low-calorie meals are prepared as a dry powder formulation. The AN-PEP and placebo are liquid formulations. All ingredients in the formulation are of food-grade quality. All formulations are produced in a food-grade facility at DSM Food Specialties. All formulations are prepared according to instructions that focus on quality. All ingredients are dry mixed and filled out in bags that are sealed airtight. Any microbial contamination will not pose a risk to the dry food because microorganisms will not be able to grow in the low water activity (<0.36) environment. The liquid formulations are provided in tubes containing benzoate as preservative, and will be stored at 2-5 ° C. All formulations of the microbial status measured. The expiry date of the formulations is issued based on the ingredient with the shortest shelf life. The ingredients in the formulations are below the acceptable daily intake established for food.

The gastric emptying rate marker octanoic acid-1-13C (Campro Scientific, Veenendaal, The Netherlands) is produced under Good Manufacturing Practice (GMP) conditions. The certificate of analysis, the material safety data sheet and the GMP statement are provided in Appendix D4. The procedure of determining gastric emptying rate with the use of octanoic acid-1-13C has also been used by our group in a recently finished human intervention study, which was previously approved by the METC AzM/UM (MEC 09-2-042 / NL27585.068.09)

PEG-3350 is used as a dilution marker for intestinal secretions. It is registered as a drug for treating chronic obstipation. A total amount of 13,5 g PEG-3350 will be infused during each test day. The recommended effective dose to treat chronic obstipation is two to three times as high (see appendix D4 ‘SPC Movicolon [Norgine]). No side effects are expected, except for a possible small effect on defecation frequency on the day of testing.

Subjects will have to spend a considerable amount of time to this intervention study. Also, they will have to discuss their medical history with the investigator, and fill in questionnaires, and suffer from the discomfort associated with catheter positioning and breath sample collection.

# Data Handling and Record Keeping

## Direct Access to Source Data/Documentation

NUTRIM will permit DFS monitoring, regulatory inspections and audits, and MEC review by providing direct access to source data/documentation.

## Storage of data and documents

Documents that are not for submission to DFS should be maintained by NUTRIM in strict confidence for maximally 15 years after study conclusion. DFS will archive all electronic raw data for a period of at least 15 years after study conclusion. DFS should inform NUTRIM when the study-related records are no longer needed and can be destroyed.

# Administrative Aspects and Reporting

## Amendments

Amendments are changes made to the research after a favourable opinion by the accredited MEC has been given. All amendments will be notified to the MEC that gave a favourable opinion.

## Annual progress report

The sponsor/investigator will submit a summary of the progress of the trial to the accredited MEC once a year according to the sample progress form provided on the website of the MEC. Information will be provided on the date of inclusion of the first subject, numbers of subjects included and numbers of subjects that have completed the trial, serious adverse events and other problems.

NUTRIM will give brief updates on the progress of the study to DFS every month.

## End of study report

The investigator will notify the accredited MEC of the end of the study within a period of 8 weeks using the study termination form as retrievable from the MEC internet site. The end of the study is defined as the last subject’s last visit.

In case the study is ended prematurely, the investigator will notify the accredited MEC, including the reasons for the premature termination.

Within one year after the end of the study, the investigator/sponsor will submit a final study report with the results of the study, including any publications/abstracts of the study, to the accredited MEC.

Until the end of the confidentiality period as mentioned in article 8 of the Agreement, being the three years following obtaining the results of the study, the Maastricht University Medical Centre authors undertake to send a copy of a proposed publication or oral presentation containing any results to the sponsor.

If the sponsor determines that the proposed publication/ presentation contains DSM-information or information which will jeopardise the protection by the sponsor of any results by intellectual property rights, and the sponsor notifies the Maastricht University Medical Centre authors of its objections within four (4) weeks after reception of the copy, the parties shall negotiate in good faith any changes to the proposed publication/presentation, without jeopardising the scientific integrity of the project, its results, NUTRIM or its researchers.

If the proposed publication/presentation contains DSM information, the sponsor has the right to require that such DSM Information shall be removed from the proposed publication/presentation.

If the proposed publication/presentation contains information which will jeopardise the protection of the sponsor of any results by intellectual property rights, the sponsor has the right to delay the proposed publication/presentation for a maximum period of three (3) months to allow the sponsor to adequately protect any results by intellectual property rights, where necessary through negotiations in good faith with the Maastricht University Medical Centre authors. This period shall commence when the sponsor has received a copy of the proposed publication/ presentation. Any prolongation of this period of delay will require the prior consent of the Medical Ethical Committee azM/UM.

In the event the four (4) week period elapses without notification from the sponsor, or (in case of permitted delay as mentioned above) if the delay period has elapsed, the Maastricht University Medical Centre authors shall be free to disclose the publication/ presentation.

In any event, the objections are deemed to be withdrawn in case the Maastricht University Medical Centre authors remove the parts of the publication/presentation objected to.

# References

Beglinger C, Fried M, Whitehouse I, Jansen JB, Lamers CB, Gyr K. Pancreatic enzyme response to a liquid meal and to hormonal stimulation. Correlation with plasma secretin and cholecystokinin levels.J. Clin. Invest. 1985:75 (May): 1471-1476.

Cerf-Bensussan N, Matysiak-Budnik T, Cellier C, Heyman M. Oral proteases: a new approach to managing coeliac disease. Gut. 2007 Feb;56(2):157-60.

Kuwabara T. Characterization of a prolyl endopeptidase from spinach thylakoids. FEBS Lett. 1992 Mar 30;300(2):127-30.

Lam WF, Gielkens AJ, Coenraad M, Souverijn JHM, Lamers CBHW, Masclee AAM. Effect of insulin and glucose on basal and cholecystokinin-stimulated exocrine pancreatic secretion in humans. Pancreas; 18(3): 252-258.

Mitea C, Havenaar R, Drijfhout JW, Edens L, Dekking L, Koning F. Efficient degradation of gluten by a prolyl endoprotease in a gastrointestinal model: implications for coeliac disease. Gut. 2008;57(1):25-32.

Stepniak D, Spaenij-Dekking L, Mitea C, Moester M, de Ru A, Baak-Pablo R, van Veelen P, Edens L, Koning F. Highly efficient gluten degradation with a newly identified prolyl endoprotease: implications for celiac disease. Am J Physiol Gastrointest Liver Physiol. 2006;291(4):G621-9.

Sattar AK, Yamamoto N, Yoshimoto T, Tsuru D. Purification and characterization of an extracellular prolyl endopeptidase from Agaricus bisporus. J Biochem. 1990 Feb;107(2):256-61.

Tack GJ, van de Water JM, Bruins MJ, et al. Co-ingestion of a gluten-degrading enzyme together with gluten in coeliac patients: a proof of concept study. Paper in submission.

Vu MK, Veek van der PPJ, Frölich M, Souverijn JHM, Biemond I, Lamers CBHW, Masclee AAM. Does jejunal feeding activate exocrine pancreatic secretion? Eur J Clin Invest 1999; 29, 1053-1059
